# The Functions and Disorders of the Reproductive Organs in Childhood, Youth, Adult Age, and Advanced Life Considered in Their Physiological, Social, and Moral Relations

**Published:** 1864-11

**Authors:** 


The Functions and Disorders of the Reproductive Organs in Childhood, Youth, Adult Age, and Advanced Life Considered in their Physiological, Social, and Moral Relations. By William Acton, M.R.C.S., Late Surgeon to the Islington Dispensary; and formerly Externe to the Venerial Hospitals, Paris; Fellow of the Royal Med. and Chirurg. and Statistical Societies, &c. &c. From the last London Edition. Philadelphia: Lindsay & Blakiston. 1864.

This is a full-sized octavo volume of 269 pages, and contains much interesting and important matter. We shall refer to it again at a future time.

